# Hospice care for 86 year old male with recurrent breast cancer: a case report

**DOI:** 10.4076/1757-1626-2-8357

**Published:** 2009-06-25

**Authors:** Maria Rosaida Gonzalez, Claudia Dolores Marcelo, Matthew Stringer

**Affiliations:** 1 Department of Medical Education, Florida Hospital East Orlando Medical Plaza7975 Lake Underhill Road, Suite 200, Orlando, FL 32822USA; 2 College of Osteopathic Medicine, Nova Southeastern University3200 South University Drive, 4^th^ floor, Fort Lauderdale, FL 33328USA

## Abstract

Terminal illness poses a tremendous challenge to patients, their families and their health care providers. It is often difficult to determine when hospice is appropriate. Timely referrals are beneficial to both patient and caregivers as a way to offer improved care and support at end of life; when multiple, complicated, co-morbid states affect care. This is especially true when a patient’s psychosocial background would otherwise make it difficult to ensure proper comfort and quality of life. In this report, we present an 86 year old man with a history of right-sided breast cancer, bipolar disorder and dementia. Patient is 20 years status-post a total mastectomy. He declined adjuvant radiation, chemotherapy, and hormone therapy, and did not pursue any further medical follow-up. Patient now presented with a rapidly enlarging, ulcerating right anterior chest wall tumor. Surgical biopsy revealed recurrent infiltrating ductal carcinoma of the breast. Patient was started on tamoxifen and palliative radiation. An oncology evaluation determined that he is not a candidate for curative treatment. Patient’s primary caregiver (wife) concurrently suffers from dementia, one son is Bipolar, and the second son is out of state. A full geriatric assessment concluded that based on the patient’s medical and socioeconomic history, he is an ideal candidate for hospice. With the family’s consent, he was enrolled in one of our local hospice organizations. Currently, he is in a skilled nursing facility with hospice.

## Introduction

“You matter because you are you. You matter until the last moment of your life, and we will do all we can, not only to help you to die peacefully, but also to live until you die.”

Dr. Cicely Mary Saunders, South London, England. Founder of the Modern Hospice Movement.

According to the World Health Organization (WHO), palliative care is an approach that improves the quality of life of patients and their families facing the problem associated with life-threatening illness. This is accomplished through the prevention and relief of suffering by means of early identification, impeccable assessment and timely treatment of pain and other problems including physical, psychosocial and spiritual ones.

Hospice, as part of the palliative care continuum, is a special program of care designed to provide comfort and support to patients and their families when a life-limiting illness no longer responds to cure-oriented treatments. Hospice addresses all symptoms of a disease with a special emphasis on controlling a patient’s pain and discomfort. Additionally, it supports the well being of primary caregivers (usually family members) and provides bereavement care for survivors [1]. It is an option for any patient who has a terminal illness and an estimated prognosis of less than six months of life. Frail, elderly patients with progressive functional and cognitive impairment along with chronic conditions in lieu of a terminal illness should also be evaluated and referred to hospice when appropriate [2].

The purpose of presenting the case of an 86 year old male with recurrent breast cancer is to highlight the role primary care physicians have in providing end of life care, which includes recognizing the need for and recommending hospice care when suitable. In this situation, hospice referral was recommended to patient and family based on three separate, but interrelated conditions: 1) patient was diagnosed with recurrent breast cancer and is not a candidate for curative treatment; 2) patient has bipolar disorder and suffers from severe cognitive decline with dementia; and 3) patient’s main caregivers are unable to care for patient due to their own debilitating health status.

It is important to note that male breast cancer is uncommon, and this case of recurrent male breast cancer is not only rare, but also unique given the patient’s biopsychosocial background. The lifetime risk of getting male breast cancer is about 1/10th of 1% (1 in 1,000), and it is 100 times less common in men than in women [3]. The most significant risk factors are inherited genetic mutations (BRCA2 gene), a family history of breast cancer, and conditions resulting in excess estrogen (Klinefelter’s disease, liver disease). Survival rates are near 100% if detected at Stage 0 or I, and 24% if Stage IV. In our patient, recurrence was most likely secondary to lack of adjuvant therapies after initial mastectomy and lack of medical follow-up, which in turn was influenced by the patient’s psychiatric history and progressive dementia along with the caregiver’s inability to recognize the patient’s symptoms due to her own advanced age and health limitations.

## Case presentation

This is an 86-year-old Caucasian male with a 20 year history of right-sided breast cancer. He presented to the emergency department with complaints of an ulcerated, hemorrhaging right anterior chest mass. Patient was noted to be a poor historian with a history of dementia. He was alert and oriented only to person and time.

Pertinent surgical history included a right total mastectomy 20 years ago. Patient did not receive adjuvant radiation, chemotherapy, or hormone therapy post-operatively. He also refused further medical care. Per the family, the patient developed a mass on his anterior chest wall approximately two years ago. The mass progressed in size, began to ulcerate and eventually the patient was brought to the emergency department after it began to bleed.

The patient’s past medical history also includes bipolar disorder, dementia, and hypertension. His surgical history is significant for a right-sided mastectomy. Current medications include: Lithium 300 mg daily. He has no known drug allergies. His mother had colon cancer, otherwise, no family history of breast cancer. Patient is married and has been retired since the age of 62. His wife suffers from new onset dementia, atrial fibrillation, chronic anemia, chronic kidney disease one son has history of bipolar disease, but is functional, and helps his parents with some of the activities of daily living. His second son lives out of state. The family has the support of a family-friend who has been helping the family through the years. Patient has a remote history of smoking cigarettes and cigars, but denies use of alcohol or recreational drugs.

**Figure 1.**
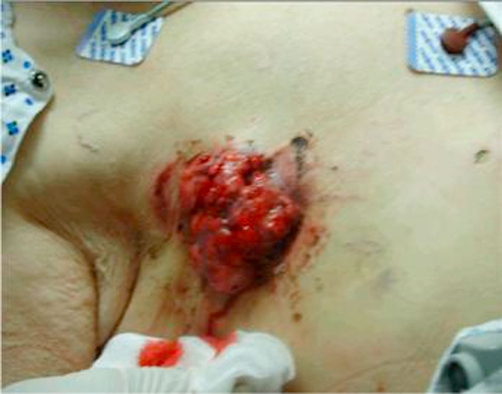
Recurrent male breast cancer: Pre-palliative treatment. Right anterior chest wall tumor.

**Figure 2.**
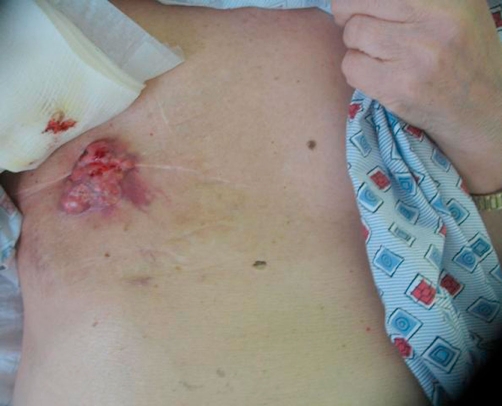
Recurrent male breast cancer: Status-post one palliative radiation treatment. Right anterior chest wall.

Vital signs on day of admission were normal with a temperature of 97.4°F, pulse of 80, respiratory rate of 14, pulse oximetry of 100% on room air. Blood pressure was elevated at 162/88. Examination findings were remarkable for a right-sided pedunculated 8 cm × 7 cm mass with a cauliflower-like appearance. The mass was ulcerated, erythematous, malodorous, and with scant bleeding (Figures 1 & 2).

Laboratory studies showed a white blood cell count 6,500, hemoglobin 12.4, hematocrit 36.2 and platelet count 178,000. Chemistry profile revealed a creatinine of 1.72 and glucose 106. The remainder was within normal limits.

An initial chest X-ray revealed findings suspicious for prior right-sided mastectomy, as well as a prominent superior mediastinum suggesting a nonspecific mediastinal mass or adenopathy. A CT chest showed a soft tissue mass in right chest wall measuring 5.2 × 2.75 × 5 cm with post-operative changes of the right axilla. CT Head showed no intracranial hemorrhage, mass effect or shift. CT abdomen and pelvis confirmed the anterior chest wall mass, but there was no evidence of metastatic deposits within the abdomen or pelvis. A bone scan also ruled out metastatic disease.

An incisional biopsy of the right breast mass was performed. Pathology returned features consistent with recurrent moderately differentiated duct carcinoma of the breast with ulceration of overlying epithelium. Nottingham combined histologic grade was T3N3M1. Breast studies returned estrogen receptor positive and progesterone receptor positive. HER-2/neu by FISH methodology was normal.

Patient was discharged to a skilled nursing facility and started on hormone therapy with tamoxifen 20 mg daily with one course of palliative radiation. An oncology evaluation determined that patient is not a candidate for curative treatment. During an initial meeting with the family at the nursing home, the family verbalized concerns over the patient’s primary caregiver’s well being and state of health and stated she was not able to properly care for her husband. Hospice care was recommended, and family agreed. Currently the patient is in a skilled nursing facility with hospice. His wife has been admitted to the same nursing facility due to general deconditioning.

## Discussion

Palliative medicine is the specialty of medicine that focuses on the quality of life of patients with advanced disease and their families. It encompasses physical, psychological, social, and spiritual aspects of end of life care. Interdisciplinary teams, therefore, deliver palliative care. The teams may include physicians, nurses, social workers, chaplains, complementary therapy practitioners, pharmacists, and volunteers. Whenever possible, they assist in the delivery of coordinated services at all sites of care (home, physicians’ offices, hospitals, and nursing homes).

Palliative medicine accompanies disease-oriented treatment. It offers relief of suffering even when the disease processes cannot be slowed, and it extends past the death of the patient to provide comfort and care to the bereaved family [4]. Palliative care is applicable to patients with significant disease, but whose prognosis is too unsure to consider hospice. When life expectancy is limited to months, rather than years, hospice is the optimal recommendation in the palliative care continuum. It is at this point, when the role of primary care physicians becomes crucial. Because of the close relationship they develop with patients and their families, primary care physicians should be able to recognize the need for and make hospice recommendations when deemed appropriate. Though it can be difficult to determine when to make a hospice referral, guidelines from Palliative Care Organizations are available to aid in the decision process [5]. Timely referrals offer more support and improved care to patients. Due to the strong relationships primary physicians are able to establish with their patients and families, they are in a unique position to emphasize quality of life and reduce medicalization.

In this particular case, after careful oncology and geriatric evaluations, the patient was not considered to be a good candidate for chemotherapy or other curative treatment. Taking into consideration the goals of care of the family, the patient’s preference, and the multiple co-morbidities of the patient, it was determined to enroll the patient to one of our hospice organizations.

## Conclusions

This case illustrates the complexity of delivering care to geriatric patients. In this case, the recurrence of breast cancer in a patient that declined medical care for years was complicated by several factors. Curative treatment was not feasible based on a geriatric evaluation, which considered the patient’s severe cognitive decline, advanced age of the patient’s caregiver, and the expectations of the patient and his family. After careful discussion between the primary care physician and the family, hospice care was initiated.

Patient was started on morphine sulfate as needed for pain control, lithium carbonate to control his bipolar illness, and bisacodyl suppositories as a preventive measure to avoid opiod induced constipation. Primary care physician visits the patient weekly to make adjustments to the medications and handle any new issues. A social worker helps address family dynamic issues and checks on the patient’s spouse and son’s well being. A chaplain addresses spiritual concerns with patient and family.

This report outlines the importance of a good communication between the patient-family unit and the physician(s) when establishing the goals of care. If appropriate, palliative care and its continuum at the end of life (also known as hospice) improve greatly the patient’s quality of life by giving the patient a sense of control and dignity over his symptoms. As far as the families of those patients, this approach will provide social, spiritual and bereavement services to the surviving family members making the transition much easier for the family and facilitating the overall well being of the surviving family members.

## Abbreviations

CT: Computed tomography
HER-2/neu: Human Epidermal growth factor Receptor 2
FISH: Fluorescence in situ hybridization
